# Correlation Analysis of Root Physiological Indicators and Metabolites of *Medicago sativa* During Overwintering Period

**DOI:** 10.3390/biology15212000

**Published:** 2026-11-04

**Authors:** Xiaolong Wang, Yue Wu, Hua Chai, Shasha Li, Wei Li, Zhao Yang

**Affiliations:** Branch of Animal Husbandry and Veterinary of Heilongjiang Academy of Agricultural Sciences, Qiqihar 161005, China; 17623409211@163.com (Y.W.); chaihua101@sina.com (H.C.); liheshasha@126.com (S.L.); tm2510@163.com (W.L.)

**Keywords:** *Medicago sativa*, cold tolerance, metabolome, physiological indicators, correlation analysis

## Abstract

*Medicago sativa* is a high-yielding and high-protein forage crop with the largest cultivation area worldwide. However, winter freezing injury severely limits its growth in high-latitude regions of Northeast China, particularly affecting the root system, which acts as a vital functional tissue for cold resistance. To date, the physiological characteristics and metabolic responses of *M. sativa* roots to low temperatures remain unclear. In this study, roots of the cold-tolerant cultivar ‘Longmu801′ and the cold-sensitive cultivar ‘Sardi’ were compared during the overwintering period. Root physiological indicators were measured, and metabolomic profiling was performed. The results showed that the contents of glutathione (GSH), sucrose (SUC), soluble sugar (SS), and proline (Pro) in Longmu801 roots were markedly higher than those in Sardi, while peroxidase and superoxide dismutase activities and the contents of H_2_O_2_ and malondialdehyde (MDA) were significantly lower (*p* < 0.05). Metabolomic analysis identified 304 differentially accumulated metabolites (DAMs). Correlation analysis between DAMs and physiological indicators showed that DAMs in the MEblue module were positively and significantly correlated with glutathione, proline, and soluble sugar (*p* < 0.05). These DAMs were mainly associated with pathways including arginine and proline metabolism, glutathione metabolism, the tricarboxylic acid cycle, and pyruvate metabolism. Four candidate DAMs related to cold tolerance were further identified, namely spermidine, N-acetyl-L-glutamate 5-semialdehyde, citric acid, and malic acid. These findings provide a theoretical basis and candidate metabolic markers for breeding cold-tolerance *M. sativa* cultivars and improving overwintering production in high-latitude cold regions.

## Simple Summary

*Medicago sativa* is a key forage crop, but severe winter conditions in cold regions damage its roots and reduce yields. To elucidate why cold-tolerant cultivars overwinter better than cold-sensitive ones, this study examined root responses to cold at the physiological and metabolic levels. Cold-tolerant cultivars maintained higher levels of protective substances, including sugars, proline, and glutathione, and showed less cellular damage. The study further identified four candidate metabolites critical to winter survival: spermidine, N-acetyl-L-glutamate 5-semialdehyde, citric acid, and malic acid. Two of these protect cells from damage, whereas the other two supply energy for cold tolerance. These findings provide targets for breeding *Medicago sativa* cultivars with improved winter hardiness, helping farmers in cold climates secure a stable supply of high-quality forage and supporting sustainable meat and dairy production.

## 1. Introduction

Plants are continuously exposed to diverse abiotic stressors, including cold, drought, and salinity, throughout their life cycle. Among these stresses, low-temperature stress alone causes approximately 40% of annual production losses in temperate-zone crops [1]. In recent years, the increasing frequency of global climate disasters, accompanied by recurrent extreme winter cold events, has exposed extensive vegetation to a heightened risk of freezing injury [2]. Low-temperature stress not only inhibits plant growth and development but also disrupts physiological and metabolic homeostasis, potentially leading to plant death under severe conditions [3]. Therefore, elucidating the physiological and metabolic responses of plants to low-temperature stress is of great significance for addressing the adverse impacts of future extreme cold events, improving crop cold tolerance, and ensuring national food security [4].

As the most widely distributed and economically valuable leguminous forage crop worldwide, *M. sativa* occupies a dominant position in China’s high-quality forage production [5,6,7]. Heilongjiang Province is an important livestock production base in China, with a strong demand for high-quality *M. sativa* as forage for herbivores, including dairy cattle, beef cattle, and mutton sheep. Located at high latitudes, the province frequently experiences extremely low winter temperatures that easily induce freezing injury to *M. sativa* roots [8]. Therefore, successful overwintering of *M. sativa* has become a major technical challenge restricting the expansion of the regional *M. sativa* industry. Under these conditions, *M. sativa* cultivars suitable for local cultivation need not only high yield and superior quality but also strong cold tolerance. Although several *M. sativa* varieties have been introduced to Heilongjiang from the United States, Canada, Australia, and other countries in recent years, many fail to adapt to the severe local winters, resulting in reduced overwintering survival and substantial yield losses [9,10,11]. The root system acts as a critical functional organ enabling *M. sativa* to survive winter cold, and its nutrient storage capacity contributes to plant cold tolerance, yield and quality. In response to low-temperature stress, plants activate a series of defense responses to attenuate freezing injury. On the one hand, plants accumulate osmoprotectants, such as soluble sugar (SS), proline (Pro), and glutathione (GSH), to maintain cellular osmotic homeostasis [12]. On the other hand, plants boost the activities of antioxidant enzymes, including superoxide dismutase (SOD), peroxidase (POD), and glutathione S-transferase (GST), to alleviate cell membrane damage induced by excessive reactive oxygen species (ROS) accumulation (H_2_O_2_ and O_2_•^-^) [13].

However, physiological indicators alone cannot fully evaluate *M. sativa* cold tolerance; therefore, metabolite responses should also be considered to further characterize root traits associated with cold tolerance. Under low-temperature conditions, plant metabolites generally exhibit three major variation patterns: (1) Amino acids: the contents of arginine, Pro, and other amino acids generally rise, indicating that *M. sativa* may enhance amino acid biosynthesis to cope with low-temperature stress [14]. (2) Organic acids: the levels of citric acid, malic acid, and other organic acids increase, which may be associated with energy metabolism and carbohydrate transformation under cold conditions [15]. (3) Sugars: the concentrations of glucose, fructose, and other saccharides may decline, suggesting that *M. sativa* utilizes sugars as energy substrates to sustain essential physiological activities under low-temperature stress [16]. The accumulation of amino acids and organic acids is closely associated with cold-tolerance-related metabolic processes, while changes in sugar levels may reflect energy allocation under low-temperature stress [17]. The metabolite variation patterns provide insights into potential metabolic strategies adopted by *M. sativa* in response to winter low-temperature stress.

Currently, the relationships between physiological indicators and metabolites in overwintering *M. sativa* roots remain unclear. Therefore, this study used overwintering *M. sativa* roots as research material and integrated physiological indicators with differential metabolite profiling to characterize the physiological and metabolic responses of *M. sativa* roots under low-temperature stress, aiming to offer a theoretical basis for the genetic enhancement of cold-tolerant *M. sativa* in high-latitude agroecosystems.

## 2. Materials and Methods

### 2.1. Experimental Design

The cold-tolerant cultivar ‘Longmu801′ (fall dormancy class 1, supplied by the Heilongjiang Academy of Agricultural Sciences) and the cold-sensitive cultivar ‘Sardi’ (fall dormancy class 7, provided by Barenbrug Tianjin International Forage Co., Ltd., China) were selected as experimental materials. Seed germination experiments were conducted at the Key Laboratory of Forage Breeding of Heilongjiang Province, Qiqihar, China, from 6 to 9 May 2025. One hundred full-sized seeds were placed in 12 cm Petri dishes containing two layers of filter paper as the substrate, and 10 mL of distilled water was added to each dish. Petri dishes were incubated in an illuminated growth chamber under conditions of 25 °C, 80% relative humidity, 15,000 lux light intensity, and a 12 h light/dark cycle. Petri dish germination served not only as a pre-germination step before transplanting but also as a means of selecting seedlings with uniform germination vigor. Because seeds germinated at different times, with differences of 2–3 days, direct sowing could result in uneven growth. Therefore, seeds were germinated first, and only uniform seedlings were transplanted. Three days after germination, 10 uniformly grown seedlings were transplanted into experimental pots (29 cm × 25 cm) containing 10 kg of field soil per pot. The plants were grown in pots filled with field soil under natural field conditions rather than being transplanted directly into the ground. The basic physicochemical properties of the soil were as follows: organic matter, 29.40 g·kg^−1^; pH 8.2; total nitrogen, 1.72 g·kg^−1^; available phosphorus, 42.27 mg·kg^−1^; and available potassium, 249.43 mg·kg^−1^. The experiment commenced on May 10. Plants were watered every 3 days. When seedlings developed 5–6 ternate compound leaves, the plants were thinned to retain eight plants per pot, and soil water content was maintained at 75%. The seedlings were then cultivated under natural field conditions in Qiqihar City, Heilongjiang Province, China (123°24′ E, 47°9′ N), where the minimum winter temperature can reach −39 °C, and the annual accumulated temperature above 10 °C is 2722 °C.

Each treatment × cultivar combination consisted of six independent pots (*n* = 6), with one pot per replicate. Roots from the eight plants within each pot were pooled to form one composite sample. The experimental unit and biological replicate were the pot, not the individual plant. This study was a pot experiment conducted under natural field conditions, not a field-plot experiment. Therefore, no micro-plot layout was established, and plants were not transplanted directly into the ground. The pots were randomly arranged in the field to minimize positional effects.

A flowering-period reference group (CK) and an overwintering-period group (YD) were established from the field-grown pot experiment. Both CK and YD plants were cultivated in pots under natural field conditions, and no growth chamber was used after transplanting. The CK group was sampled at the flowering period, whereas the YD group was sampled during natural overwintering. Environmental conditions were monitored rather than controlled. Air temperature was recorded at 30 min intervals using shielded, factory-calibrated iButton Thermochron data loggers (DS1922L, Maxim Integrated, San Jose, CA, USA) installed at plant canopy height (approximately 20 cm above ground) in each treatment block. Photosynthetic photon flux density was recorded at 1 min intervals using a quantum sensor (LI-190R, LI-COR Biosciences, Lincoln, NE, USA) connected to a data logger (CR1000X, Campbell Scientific, Logan, UT, USA). Photoperiod was calculated as the duration during which photosynthetic photon flux density exceeded 5 μmol m^−2^ s^−1^. The study covered one growing season and one natural overwintering cycle, from May 2025 to May 2026. Given the high environmental variability under field conditions, this work should be regarded as a preliminary study.

During the CK flowering sampling period, the mean daytime and nighttime air temperatures were 25 °C and 16 °C, respectively, and the natural photoperiod was 14 h light/10 h dark. The CK subgroups included (a) CK_Longmu801 and (b) CK_Sardi. During the YD overwintering sampling period, the mean daytime and nighttime air temperatures were −15 °C and −25 °C, respectively, and the natural photoperiod was 10 h light/14 h dark. The YD subgroups included (c) YD_Longmu801 and (d) YD_Sardi. No micro-plot arrangement was established. This study was conducted as a pot experiment under natural field conditions, with the pot considered the experimental unit. Each sampling period × cultivar combination consisted of six replicate pots, with eight uniform plants retained in each pot.

Root tissues of *M. sativa* were harvested in Qiqihar City, Heilongjiang Province, China, from the CK group at the flowering period (20 June) and from the YD group during the overwintering period (20 December). The root crowns were rinsed with deionized water, and selected root samples were used for physiological measurements. These measurements were performed at the Key Laboratory of Forage Breeding of Heilongjiang Province, Qiqihar, China, from June to December 2025. Fresh root samples were ground in liquid nitrogen. Approximately 0.1 g of fresh root tissue was extracted with the corresponding buffer or solvent, homogenized in an ice bath, and centrifuged at 4 °C (12,000 rpm for 15 min). The supernatant was collected for subsequent assays.

### 2.2. Physiological Measurements

The following physiological indices were measured: (1) SOD activity was measured by the nitroblue tetrazolium (NBT) photochemical reduction method. The reaction mixture contained enzyme extract, phosphate buffer (50 mM, pH 7.8), methionine, NBT, EDTA-Na_2_, and riboflavin. Absorbance was measured at 560 nm. One unit of SOD activity was defined as the amount of enzyme required to inhibit NBT photoreduction by 50%. The results were expressed as U g^−1^ FW [18]. (2) POD activity was measured by the guaiacol oxidation method. The reaction mixture contained enzyme extract, phosphate buffer (50 mM, pH 7.0), guaiacol, and H_2_O_2_. Absorbance was measured at 470 nm. One unit of POD activity was defined as an increase of 0.01 absorbance units per minute. The results were expressed as U g^−1^ FW min^−1^ [18]. (3) SS content was determined using the anthrone colorimetric method after extraction with 80% ethanol. Absorbance was measured at 620 nm, with glucose as the standard. The results were expressed as mg g^−1^ FW [19]. (4) Pro content was extracted with 3% sulfosalicylic acid and measured by the acidic ninhydrin method. Absorbance was measured at 520 nm, and the content was calculated using a proline standard curve. The results were expressed as mg g^−1^ FW [20]. (5) GSH content was determined using the 5,5′-Dithiobis (2-nitrobenzoic acid) method after extraction with 5% trichloroacetic acid (TCA). Absorbance was measured at 412 nm using GSH as the standard. The results were expressed as μmol g^−1^ FW [21]. (6) MDA content was determined using the thiobarbituric acid method after extraction with 10% TCA. Absorbance was measured at 532 and 600 nm, and the MDA content was calculated using the extinction coefficient after subtracting nonspecific absorbance. The results were expressed as μmol g^−1^ FW [22]. (7) SUC was extracted with 80% ethanol, and its content was measured by the resorcinol method. Absorbance was measured at 480 nm using sucrose as the standard. The results were expressed as mg g^−1^ FW [23]. (8) H_2_O_2_ was extracted with cold acetone or TCA, and its content was measured by the xylenol orange method or a commercial H_2_O_2_ assay kit (Thermo Fisher Scientific, Madison, WI, USA) according to the manufacturer’s instructions. Absorbance was measured at the specified wavelength (560 nm), and H_2_O_2_ content was calculated using a standard curve. The results were expressed as μmol g^−1^ FW [24]. (9) O_2_•^-^ content was measured by the NBT reduction method. Samples were incubated in phosphate buffer containing NBT and NaN_3_, and absorbance was measured at 580 nm. The O_2_•^-^ level was calculated after subtracting the blank value and expressed as μmol g^−1^ FW or as an increase in absorbance [25].

### 2.3. Metabolomic Profiling and Metabolite Identification

Metabolomic profiling and analysis were performed on the sequencing and analysis platform of Shanghai Majorbio Bio-pharm Technology Co., Ltd., Shanghai, China, from January to July 2026. Root crown tissue (100 mg) from *M. sativa* was ground in liquid nitrogen and transferred into EP tubes containing 500 μL of 80% aqueous methanol. After zing, the samples were incubated on ice for 5 min and centrifuged at 4 °C for 20 min. The supernatants were diluted with water to obtain a final methanol concentration of 53%, followed by another centrifugation at 4 °C for 20 min. The extracted samples were analyzed using a Thermo Fisher Scientific UHPLC-Q Exactive HF-X (Thermo Fisher Scientific, Bremen, Germany) system (ultra-high-performance liquid chromatography coupled with Fourier transform mass spectrometry) equipped with an ACQUITY UPLC HSS T3 column (100 mm × 2.1 mm i.d., 1.8 µm; Waters, Milford, MA, USA). The column temperature was maintained at 40 °C. Mobile phase A consisted of water/acetonitrile (95:5, *v*/*v*) containing 0.1% formic acid, whereas mobile phase B consisted of acetonitrile/isopropanol/water (47.5:47.5:5, *v*/*v*/*v*) containing 0.1% formic acid. The gradient programs were as follows: positive mode (time, 0, 3, 4.5, 5, 6.3, 6.4, 8 min; B%, 0, 20, 35, 100, 100, 0, 0) and negative mode (time, 0, 1.5, 2, 4.5, 5, 6.3, 6.4, 8 min; B%, 5, 10, 30, 100, 100, 0, 0). The flow rate was 400 μL/min, and the injection volume was 3 μL. Mass spectrometric data were acquired in positive and negative ion modes. The MS1 mass scan range was *m*/*z* 70–1050, and MS/MS data were collected using data-dependent acquisition mode. The ion spray voltages were 3500 V in positive mode and −3000 V in negative mode. The sheath gas and auxiliary heating gas were set at 50 and 13 arb, respectively, and the ion source heater temperature was 450 °C. Stepped collision energies were set at 20, 40, and 60 V. Quality control (QC) samples were prepared by mixing equal volumes of supernatants from all samples and were injected once every 12 samples. Blank samples were prepared by replacing the sample matrix with extraction solvent and were processed using the same procedure. Solvent blanks and extraction blanks (prepared without plant material but processed identically to the samples) were injected to identify background contaminants and carryover. Peaks detected in blanks with an intensity ≥30% of that in the biological samples were flagged as potential contaminants and excluded. Metabolite annotations were assigned MSI confidence levels. The four candidate key metabolites were putatively annotated, and none was confirmed with authentic standards. Spermidine, citric acid, and malic acid were assigned MSI Level 2, whereas N-acetyl-L-glutamate 5-semialdehyde (NAGSA) was assigned MSI Level 3 and explicitly labeled as a tentative annotation. None of the four candidate key metabolites were detected in the blank controls. For the principal metabolites, the precursor *m*/*z*, adduct type, retention time, diagnostic MS/MS fragments, mass error, database/library match, authentic-standard status, MSI confidence level, and blank-control results are provided in Supplementary Table S1.

### 2.4. Principal Component Analysis (PCA) and Orthogonal Partial Least Squares Discriminant Analysis (OPLS-DA)

Metabolomic data are multivariate datasets containing multiple metabolite variables. PCA was performed to reduce data dimensionality, generate principal components, classify samples based on their similarity, and visualize overall metabolic variation among samples. Data processing was conducted using MetaX software (version 1.6.0), and PCA was performed using all samples, including QC samples. The tight clustering of QC samples in PCA plots indicated good analytical reproducibility and data quality.

OPLS-DA was performed to evaluate the separation between sample groups. OPLS-DA models were constructed for each pairwise comparison using metabolite abundance data from the two groups.

### 2.5. Kyoto Encyclopedia of Genes and Genomes (KEGG) Enrichment Analysis of Differentially Accumulated Metabolites (DAMs)

DAMs were screened based on variable importance in projection (VIP) scores, fold change (FC), and *p* values. The *p* values were calculated using Student’s *t*-test to evaluate the statistical significance of differences in metabolite abundance between groups. The screening criteria were set as VIP > 1.0, FC > 1.2 or FC < 0.83, and *p* < 0.05. Venn diagrams were generated to compare shared and unique DAMs among different groups during overwintering. The complete quantitative dataset for the 304 DAMs is provided in Supplementary Table S2. KEGG pathway enrichment analysis was performed using Scipy (version 1.0.0). The background set consisted of all detected metabolites with KEGG compound IDs, whereas DAMs were used as the query set. Metabolites were mapped to the KEGG PATHWAY database based on KEGG compound IDs, with species annotation according to *M. sativa* reference pathways. Pathway enrichment significance was assessed by the hypergeometric test. Enrichment factors were calculated as the ratio of observed to expected metabolite numbers. *p* values were adjusted for multiple testing using the Benjamini–Hochberg method, and pathways with adjusted *p* < 0.05 were considered significantly enriched.

### 2.6. Data Analysis

Statistical analyses were performed using SAS 9.0 software (SAS Institute Inc., Cary, NC, USA). The data from the four root sample groups followed a normal distribution, satisfying the assumptions for parametric tests. Differences among multiple groups were assessed using one-way analysis of variance, followed by Duncan’s multiple range test. A *p*-value < 0.05 was considered statistically significant.

Metabolomic profiling and metabolite identification were performed using Progenesis QI v3.0 (Waters Corporation, Milford, MA, USA) for peak extraction, alignment, and filtering. Metabolites were identified by matching retention time, accurate mass, isotope pattern, and MS/MS fragments against public metabolite databases including HMDB (http://www.hmdb.ca/) (accessed on 10 October 2026), METLIN (https://metlin.scripps.edu/) (accessed on 15 October 2026), and the Majorbio in-house database. The identification criteria were a mass error of ≤10 ppm and an MS/MS match score of ≥35.

For OPLS-DA, each comparison included six biological replicates per group; therefore, twelve samples were included in each model (six biological replicates per group; *n* = 12). Leave-one-out cross-validation was performed on the entire pairwise dataset, with k = N = 12 folds. In each fold, one sample was held out as the validation set, while the remaining eleven samples (11/12 of the dataset) were used for model training. The held-out sample (1/12 of the dataset) was then predicted. This procedure was repeated twelve times, ensuring that each sample was used once as the validation set. Notably, this approach represents leave-one-out cross-validation across the entire dataset rather than six-fold cross-validation performed separately within each group. Model performance was evaluated using R^2^Y and Q^2^ values (R^2^Y = 0.995, Q^2^ = 0.985), with values closer to 1 indicating better model fit and predictive performance. Q^2^ values greater than 0.5 were generally considered acceptable. Furthermore, permutation testing with 200 permutations was performed to rigorously assess the risk of overfitting. Differential metabolites were identified based on OPLS-DA VIP > 1.0 combined with FC > 1.2 or FC < 0.83 and *p* < 0.05.

Candidate metabolites associated with cold tolerance were selected using a stepwise and reproducible procedure. Metabolites were retained when they met all of the following criteria: (1) VIP > 1.0 in the OPLS-DA model; (2) FC > 1.2 or FC < 0.83; (3) significant enrichment in KEGG pathways associated with low-temperature responses; (4) membership in the MEblue module; and (5) significant correlations with physiological indicators. Based on these criteria, four candidate metabolites were identified: spermidine, N-acetyl-L-glutamate 5-semialdehyde, malic acid, and citric acid. Due to its relatively low annotation confidence, NAGSA was labeled as a tentative candidate.

Weighted gene co-expression network analysis (WGCNA) was performed using the WGCNA R package (v1.71). Metabolite abundance data were log_2_-transformed and Z-score standardized before network construction. Samples and metabolites with excessive missing values were removed using the goodSamplesGenes (datExpr, verbose = 3) function, and outlier samples were identified and excluded based on hierarchical clustering with average linkage. Biweight midcorrelation (bicor) was used as the similarity measure to construct the adjacency matrix. The soft-thresholding power (β) was determined using the pickSoftThreshold (datExpr, powerVector = 1:20, networkType = “signed”, corFnc = “bicor”, verbose = 5) function, and the lowest β value achieving a scale-free topology fit index (R^2^) > 0.85 was selected. Co-expression modules were detected using the blockwiseModules (datExpr, power = softPower, networkType = “signed”, TOMType = “signed”, corType = “bicor”, minModuleSize = 30, mergeCutHeight = 0.25, deepSplit = 2, pamRespectsDendro = FALSE) function. Module eigengenes (MEs), defined as the first principal component of each module, were correlated with physiological traits using Pearson correlation analysis. Modules with |correlation| > 0.5 and *p* < 0.05 were considered significantly associated with physiological traits. Multiple testing across modules and traits was addressed using the Benjamini–Hochberg procedure to adjust the *p*-values, with an FDR-adjusted q < 0.05 considered statistically significant. Hub metabolites were identified based on high module membership (|MM| > 0.5) and metabolite significance (|GS| > 0.5).

## 3. Results

### 3.1. Physiological Indicators

During the overwintering period, Longmu801 roots displayed better physiological performance than Sardi roots (Figure 1A). Compared with YD_Longmu801, the contents of POD, SOD, H_2_O_2_, O_2_•^-^, and MDA in YD_Sardi roots were significantly higher (*p* < 0.05), being 1.03-, 1.17-, 1.21-, 1.19-, and 1.07-fold higher, respectively (Figure 1B,C,G,I,J). Conversely, the contents of GSH, SS, and Pro in YD_Longmu801 roots were 1.15-, 1.19-, and 1.09-fold higher, respectively, than those in YD_Sardi roots (Figure 1D–F). Additionally, the SUC content in YD_Longmu801 roots was markedly higher than that in YD_Sardi roots (*p* < 0.05, Figure 1H).

### 3.2. Assembly and Classification of Metabolomic Profiling Data

PCA score plots showed clear separation between samples collected during the flowering period (CK) and overwintering period (YD) along PC1, forming two distinct clusters with limited overlap, suggesting differences in overall metabolic profiles between the two sampling periods. In addition, YD_Longmu801 and YD_Sardi samples were separated along PC2, although YD_Sardi showed greater dispersion, suggesting cultivar-dependent differences in metabolite profiles during overwintering (Figure S1A). As shown in Figure S1B, the numbers of DAMs identified in the comparisons of YD_Longmu801 vs. CK_Longmu801, YD_Sardi vs. CK_Sardi, and YD_Longmu801 vs. YD_Sardi were 726, 756, and 304, respectively. Among these comparisons, 111, 139, and 86 DAMs were identified in the corresponding groups, while 45 DAMs were shared among the three comparisons.

**Figure 1:**
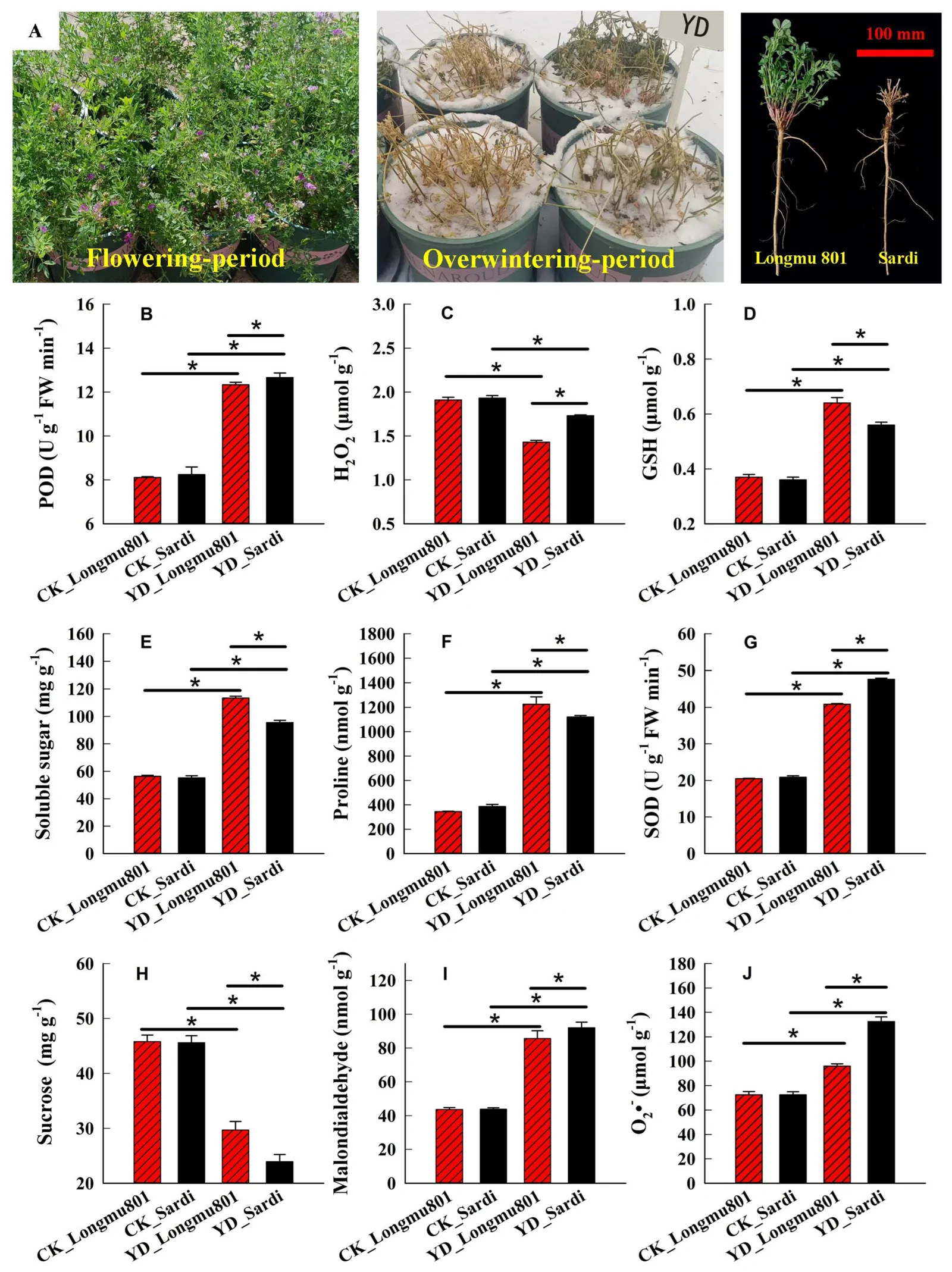
Analysis of physiological indicators of *Medicago sativa* root during the overwintering period. Root morphology during winter (**A**), POD (**B**), H_2_O_2_ (**C**), GSH (**D**), soluble sugar (**E**), proline (**F**), SOD (**G**), Sucrose (**H**), Malondialdehyde (**I**), and O_2_•^-^ (**J**). Asterisks (*) denote significant differences among groups (*p* < 0.05).

### 3.3. Functional Annotation of DAMs

A total of 1701 DAMs were identified based on OPLS-DA analysis. Among them, 297 DAMs were identified in the comparison between YD_Longmu801 and YD_Sardi, including 105 upregulated DAMs and 192 downregulated DAMs (Figure 2D). In the comparison between YD_Longmu801 and CK_Longmu801, 576 DAMs were identified, including 239 upregulated DAMs and 337 downregulated DAMs (Figure 2B). Similarly, 586 DAMs were identified between YD_Sardi and CK_Sardi, including 270 upregulated DAMs and 316 downregulated DAMs (Figure 2C).

During the overwintering period, correlation analysis between the two *Medicago sativa* varieties and DAMs revealed that YD_Longmu801 was significantly negatively correlated (*p* < 0.05) with Val-Glu, L-valine, Tyr-Gly, Asp-Leu, and pyrophosphate, whereas it was significantly positively correlated (*p* < 0.05) with NAGSA, argininosuccinic acid, spermidine, L-serine, and citric acid. In contrast, YD_Sardi exhibited a significant negative correlation (*p* < 0.05) with malic acid and NAGSA (Figure S2). Based on the correlations among DAMs, citric acid was significantly positively correlated (*p* < 0.05) with pyroglutamic acid and spermidine, with r values of 0.778 and 0.811, respectively. Malic acid showed a significant positive correlation (*p* < 0.05) with oxytetracycline, and maleic acid, with r values of 0.892 and 0.946, respectively (Figure S3).

The abundances of spermidine, argininosuccinic acid, N-(L-arginino)succinate, argininosuccinate, L-rhamnulose, L-cis-3-amino-2-pyrrolidinecarboxylic acid, and pyroglutamic acid were markedly higher in YD_Longmu801 and YD_Sardi plants than in CK_Longmu801 and CK_Sardi. Furthermore, compared with YD_Sardi, YD_Longmu801 exhibited higher relative abundances of citric acid, NAGSA, spermidine, pyroglutamic acid, argininosuccinic acid, and malic acid (Figure S4).

KEGG enrichment analysis results are shown in Figure 3. The main enriched pathways in YD_Longmu801_vs_CK_Longmu801 included ABC transporters, diterpenoid biosynthesis, and arginine and proline metabolism. The main enriched pathways in YD_Sardi_vs_CK_Sardi included ABC transporters, biosynthesis of various plant secondary metabolites, and flavonoid biosynthesis. The pathways primarily enriched in YD_Longmu801_vs_YD_Sardi included biosynthesis of cofactors, biosynthesis of various plant secondary metabolites, and tryptophan metabolism.

### 3.4. DAMs Exploratory Correlation-Based Module Analysis

An exploratory correlation-based module was constructed for the 304 DAMs identified between YD_Longmu801 and YD_Sardi, and the DAMs were divided into six modules: MEturquoise, MEblue, MEbrown, MEyellow, MEgreen, and MEgrey. These modules contained 95, 67, 46, 36, 31, and 29 DAMs, respectively (Figure 4A). The correlations between the DAM modules and physiological indicators are shown in Figure 4B. DAMs in the MEblue module were significantly positively correlated (*p* < 0.05) with POD, SOD, GSH, Pro, SS, and MDA, with corresponding r values of 0.818, 0.752, 0.897, 0.877, 0.913, and 0.790, respectively.

In the KEGG pathway analysis, spermidine was involved in glutathione metabolism, NAGSA was associated with arginine and proline metabolism, citric acid was involved in the citrate cycle and biosynthesis of cofactors, and malic acid was involved in pyruvate metabolism and glyoxylate and dicarboxylate metabolism. These four metabolites were identified as key DAMs associated with cold tolerance in *M. sativa* (Figure 5).

## 4. Discussion

Freezing temperatures represent a critical abiotic stress factor limiting the productivity of *M. sativa*. Particularly in high-latitude agroecosystems of northern China, frequent and severe winter freezing events cause root freezing damage, resulting in reduced yield and quality [26]. This study aimed to characterize the metabolic responses of *M. sativa* roots during overwintering under low-temperature stress. Through metabolomic analysis, four candidate DAMs were identified from the YD_Longmu801 vs. YD_Sardi comparison, namely spermidine, NAGSA, malic acid, and citric acid. These metabolites were significantly positively correlated with GSH, SS, and Pro, and were mainly involved in pathways related to glutathione, arginine and proline metabolism, pyruvate metabolism, and the tricarboxylic acid cycle. These results suggest that these metabolites are candidate metabolites associated with cold tolerance; however, their causal roles require further functional validation.

Spermidine (Spd) has been reported to improve plant cold tolerance, mainly by enhancing antioxidant defense and maintaining osmotic homeostasis. Spd can increase the activities of antioxidant enzymes, such as SOD and POD, scavenge low-temperature-induced ROS, and alleviate oxidative damage [27]. Meanwhile, Spd may promote the accumulation of osmoprotectants, including Pro and SS, to stabilize cell membranes. Spd accumulation has also been associated with the modulation of antioxidant systems, the elimination of excess ROS, and reductions in MDA and H_2_O_2_ levels, thereby alleviating cellular injury [28]. In this study, Spd content increased markedly in the cold-tolerant cultivar Longmu801 during overwintering. The contents of Pro, SS, and GSH in Longmu801 were significantly higher than those in the cold-sensitive cultivar Sardi, while MDA and H_2_O_2_ contents were lower. However, POD and SOD activities were also lower in Longmu801 than in Sardi. This apparent discrepancy suggests that lower antioxidant enzyme activities in the cold-tolerant cultivar may reflect a lower oxidative burden rather than weaker antioxidant defense, consistent with the lower MDA and H_2_O_2_ levels. Therefore, Spd accumulation in this study was associated with improved osmotic adjustment, higher GSH content, and reduced oxidative damage, rather than increased SOD and POD activities. Previous studies have suggested that Spd may promote the expression of the spermidine synthase gene (*SPDS*) and upregulate cold-responsive genes (e.g., *MdCBF1/2/3*), potentially contributing to cold tolerance in plants [29]. However, *SPDS* expression, WRKY27–SPDS regulation, and cold-responsive gene expression were not measured in this study. Therefore, these mechanisms should be regarded as literature-supported hypotheses rather than mechanisms directly demonstrated here. Moreover, whether Spd directly enhances SOD and POD activities in Longmu801 during overwintering requires further investigation, as our results showed lower rather than higher SOD and POD activities in this genotype. Thus, the role of Spd in cold tolerance should be interpreted cautiously and requires further functional validation. The higher levels of Spd, Pro, and GSH detected in Longmu801 under winter low-temperature conditions (Figure 5 and Figure 1) are consistent with previous reports that low temperature may induce SPDS expression in other plant systems. WRKY27 has been reported to interact with the SPDS promoter and promote its transcription, forming a proposed WRKY27-SPDS regulatory module involved in Spd biosynthesis under cold stress [30,31]. However, whether this regulatory relationship operates in *M. sativa* during overwintering remains unclear. Further studies have suggested that SPDS may regulate antioxidant systems and the accumulation of Pro and GSH, thereby contributing to cold tolerance [32]. In summary, previous studies suggest that spermidine may function beyond a simple osmoprotectant; it may couple cold signal transduction with downstream physiological responses via a regulatory cascade involving upstream transcription factors, biosynthetic enzymes, and terminal metabolites. Our data support an association between Spd accumulation and cold tolerance in *M. sativa*; however, the underlying causal mechanisms require further functional validation.

GSH plays an important role in overwintering cold tolerance. GSH is a central non-enzymatic antioxidant and redox buffer in plant cold tolerance. In this study, root GSH content was significantly higher in the cold-tolerant cultivar Longmu801 than in the cold-sensitive cultivar Sardi during overwintering (Figure 1D). This higher GSH level was accompanied by lower H_2_O_2_ and MDA contents (Figure 1C,I) and l reduced O_2_•^-^ accumulation (Figure 1J), suggesting that GSH accumulation was associated with reduced oxidative damage in Longmu801. GSH may directly scavenge ROS and act as an electron donor in the ascorbate–glutathione cycle, in which glutathione peroxidase detoxifies H_2_O_2_ and glutathione reductase regenerates GSH from GSSG using NADPH [33]. In addition, GSH contributes to glutathione S-transferase-mediated detoxification and protects protein thiols, thereby helping maintain redox homeostasis and membrane integrity under low-temperature conditions [34]. Metabolomic analysis further revealed that DAMs in the MEblue module were significantly positively correlated with GSH, Pro, and SS and were associated with glutathione metabolism, suggesting a link between GSH-related metabolic reprogramming and cold tolerance. The potential interaction between GSH and polyamine metabolism was also suggested by the annotation of glutathionyl-spermidine and related metabolites in the pathway map (Figure 5). However, only total GSH content was measured in this study. The GSH/GSSG ratio, the activities of glutathione reductase, glutathione peroxidase, and glutathione S-transferase, and the flux through the ascorbate–glutathione cycle were not determined. Therefore, the role of GSH in overwintering cold tolerance should be interpreted as an association rather than a demonstrated causal mechanism. Future studies should assess the glutathione redox state and the activities of related enzymes to clarify how GSH interacts with proline, soluble sugars, and polyamines in Longmu801 roots during winter.

NAGSA is an intermediate of arginine biosynthesis and may be involved in the connection between arginine and proline metabolism. Under low-temperature stress, L-glutamate 5-semialdehyde (GSA) is converted into NAGSA by L-glutamate-5-semialdehyde N-acetyltransferase. NAGSA is an intermediate in arginine biosynthesis (KEGG compound C01250) and is typically formed from N-acetyl-L-glutamate 5-phosphate by N-acetyl-γ-glutamyl-phosphate reductase (EC 1.2.1.38), rather than being a direct product of proline catabolism. An alternative acetyltransferase-mediated reaction (EC 2.3.1.271) that converts GSA to NAGSA has also been reported [35] and is associated with the arginine and proline metabolism pathway (map00330). In yeast, the acetyltransferase reaction can reduce the GSA/P5C pool; however, whether this reaction occurs in *M. sativa* during overwintering remains unclear. Proline plays multiple roles in plant cold responses, including osmotic adjustment and ROS scavenging, and also serves as a precursor for spermidine biosynthesis [36,37]. In this study, YD_Longmu801 accumulated high levels of NAGSA during overwintering, accompanied by markedly higher free proline and spermidine contents than those in cold-sensitive YD_Sardi. These results suggest that proline-related metabolism may contribute to cold-tolerance-associated responses and that NAGSA accumulation may reflect metabolic adjustment under cold conditions. From a literature perspective, elevated NAGSA may accompany, rather than directly regulate, increased spermidine production. Spd may contribute to membrane stability and antioxidant defense [38], consistent with the lower MDA and H_2_O_2_ levels observed in Longmu801. Alternatively, previous studies have suggested that the acetyltransferase reaction may reduce the GSA/P5C pool and potentially limit the accumulation of ROS-related precursors under cold conditions [39]. However, the relationship between GSA/P5C metabolism and ROS regulation remains to be verified in *M. sativa*. Taken together, NAGSA should be regarded as a potential indicator of arginine pathway activity associated with polyamine metabolism rather than a demonstrated cold-responsive regulatory hub. Functional validation is required to determine whether NAGSA plays an active role in linking cold responses with proline–arginine–spermidine metabolism.

Malic acid may contribute to plant cold responses through osmotic adjustment, metabolic regulation, and redox homeostasis. As a major soluble organic anion in vacuoles, malate may contribute to osmotic adjustment by working together with soluble sugars to help maintain cellular water balance under cold conditions [40]. Previous studies have demonstrated that malic acid treatment significantly reduced electrolyte leakage and malondialdehyde content while increasing SOD activity in rice seedlings under low-temperature stress, thereby improving plant cold tolerance [41]. Consistent with these findings, malic acid abundance was significantly higher in YD_Longmu801 than in YD_Sardi during overwintering, indicating an association between malate metabolism and cold tolerance. Previous studies suggest that malic acid contributes to energy production and carbon skeleton supply through the tricarboxylic acid cycle and glycolysis, and that its metabolism may be reprogrammed under cold stress to sustain basic metabolic homeostasis [42]. However, metabolic flux was not measured in this study. For redox regulation, the cytoplasmic NAD-dependent malate dehydrogenase gene (*MdcyMDH*) in apple has been reported to be induced by low-temperature stress, and its overexpression enhances the cold tolerance in transgenic plants by boosting cellular reducing capacity through ascorbic acid- and glutathione-mediated mechanisms [43,44]. Moreover, malic acid may contribute to redox homeostasis by modulating the NAD(P)H/NAD(P)^+^ ratio and alleviating cold-induced oxidative stress [42]. However, these redox-related mechanisms were not directly measured in the present study. Overall, previous studies suggest that malic acid may act as more than a metabolite involved in carbon metabolism, potentially contributing to the coordination of carbon metabolism, osmotic balance, and redox homeostasis. Our data support an association between malic acid accumulation and cold tolerance. Although manipulating malic acid metabolism may offer a potential strategy for improving crop cold tolerance, further functional validation is required.

The citric acid cycle may serve as a critical metabolic hub during overwintering in *M. sativa*. This cycle oxidizes acetyl-CoA, derived from carbohydrate, lipid, and protein catabolism, to CO_2_ and generates reducing equivalents that support ATP production [45]. Key intermediates of the cycle, including citric acid, α-ketoglutarate, and oxaloacetate, also act as precursors for numerous biosynthetic reactions, participating in the synthesis of nucleotides, amino acids, and fatty acids required for structural repair and functional preservation of roots under low-temperature stress [46]. Moreover, NADH and FADH_2_ produced during the cycle supply electrons to the electron transport chain for oxidative phosphorylation, thereby supporting ATP production and regenerating NAD^+^ and FAD. In this study, citric acid accumulation was markedly higher in YD_Longmu801 than in the cold-sensitive genotype YD_Sardi. This observation suggests that Longmu801 may have a greater ATP demand to sustain energy-intensive processes, such as maintaining cell integrity and active transport. However, ATP production, energy-consuming processes, and metabolic flux were not measured in this study; therefore, increased ATP demand cannot be confirmed by the present data. Previous studies suggest that increased energy demand may be accompanied by enhanced activities of key enzymes in the citric acid cycle, including citrate synthase and isocitrate dehydrogenase, potentially accelerating cycle flux to meet cellular energy requirements [47]. However, these enzyme activities were not measured in this study, and this mechanism remains hypothetical. In addition, citric acid may exert protective effects in *M. sativa* cv. YD_Longmu801. It may help alleviate cold-triggered oxidative stress through metal ion chelation, although its proposed role as an osmoprotectant requires further verification. Meanwhile, anaplerotic pathways, including reactions catalyzed by malic enzyme, may replenish citric acid cycle intermediates and help sustain cycle activity under increased metabolic demand. Overall, the citric acid cycle may contribute to cold tolerance in *M. sativa* by supporting ATP provision, supplying metabolic precursors, and maintaining redox and osmotic homeostasis. Our results support an association between citric acid accumulation and cold tolerance. Modulating key enzymes and pathways involved in citric acid metabolism may represent a potential strategy for improving cold tolerance in *M. sativa*, although further functional validation is required.

The limitations of this study and future research directions should be considered. This study was conducted over only one growing season and one natural overwintering cycle at a single site. Field conditions are highly variable, and observations from a single year may not capture interannual variation in temperature, snow cover, soil moisture, and other environmental factors. Therefore, this study should be regarded as a preliminary study, and the observed associations between GSH, spermidine, NAGSA, malic acid, citric acid, and cold tolerance are limited to the present dataset. In addition, ROS were quantified in bulk root tissues, whereas their spatial localization was not determined; future histochemical and fluorescent-probe analyses should address this. It should also be noted that NAGSA is a tentative annotation (MSI Level 3) and that none of the four key metabolites (spermidine, NAGSA, malic acid, and citric acid) was confirmed using authentic standards. These associations should therefore be interpreted cautiously. Further research involving multi-year and multi-site experiments, together with genetic analyses, enzyme activity assays, and metabolic flux analyses, is needed to verify the robustness and generalizability of these findings.

## 5. Conclusions

In this study, the roots of the cold-tolerant cultivar Longmu801 and the cold-sensitive cultivar Sardi were compared during overwintering. Longmu801 showed significantly higher contents of glutathione, soluble sugar, proline, and sucrose, but lower POD and SOD activities and lower H_2_O_2_, O_2_•^-^, and malondialdehyde contents than Sardi. These results suggest that Longmu801 maintains better osmotic homeostasis, greater non-enzymatic antioxidant capacity, and lower oxidative damage during winter. The lower antioxidant enzyme activities may reflect a reduced oxidative burden rather than weaker antioxidant defenses.

During overwintering, 304 DAMs were identified between Longmu801 and Sardi. DAMs in the MEblue module showed significant positive correlations with glutathione, proline, and soluble sugar levels. These DAMs were mainly annotated to pathways related to glutathione metabolism, arginine and proline metabolism, pyruvate metabolism, and the tricarboxylic acid cycle. Spermidine, NAGSA, citric acid, and malic acid were further identified as candidate metabolites associated with cold tolerance, potentially contributing to osmotic adjustment, redox regulation, polyamine metabolism, and energy metabolism. However, these metabolites should be regarded as candidates rather than established regulatory factors; their causal roles require further functional validation.

Overall, this integrated physiological and metabolomic study indicates that overwintering cold tolerance in *M. sativa* roots was associated with coordinated changes in glutathione metabolism, arginine and proline metabolism, pyruvate metabolism, and the tricarboxylic acid cycle. The identified candidate metabolites and pathways, together with the LC-MS dataset, provide a useful reference for future functional studies of cold-tolerance mechanisms in *M. sativa*. These findings provide a theoretical basis and candidate metabolic markers for breeding cold-tolerant *M. sativa* cultivars and improving overwintering production in high-latitude cold regions.

## Figures and Tables

**Figure 2:**
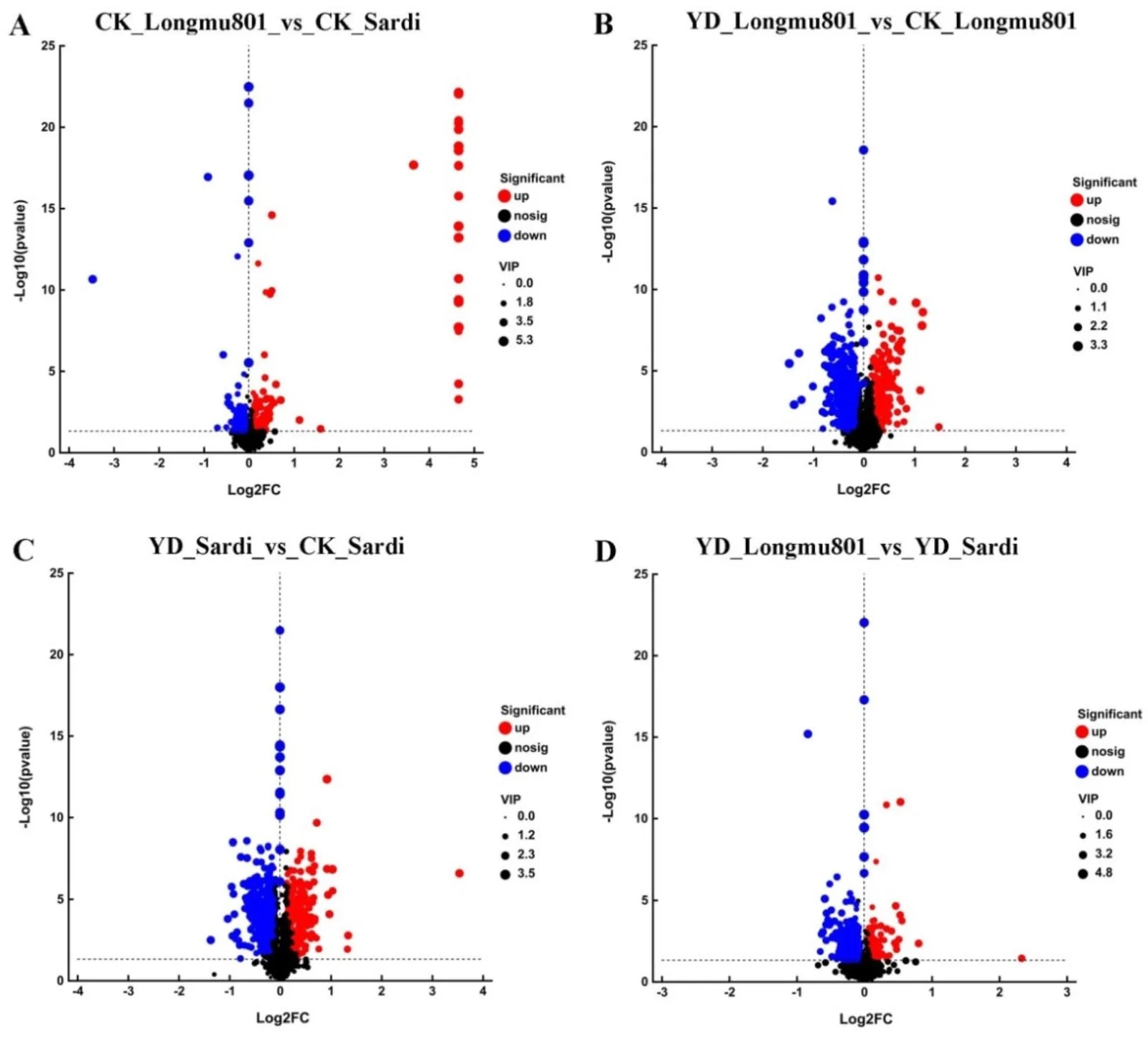
Analysis of differential metabolites in *Medicago sativa* during the overwintering period. Red denotes upregulated DAMs, blue denotes downregulated DAMs.

**Figure 3:**
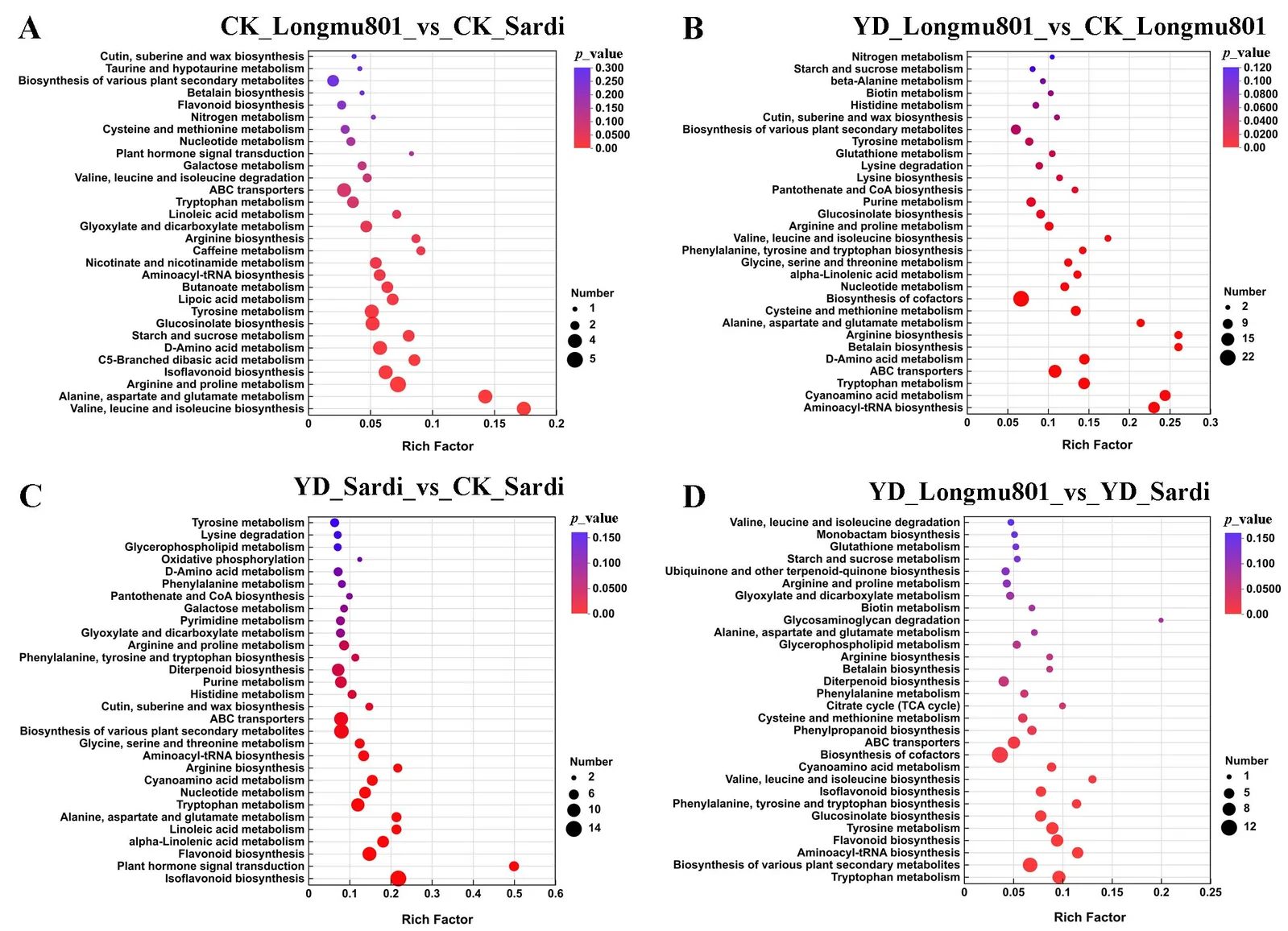
KEGG enrichment analysis of *Medicago sativa* during the overwintering period. KEGG analysis of DAMs in CK_Longmu801_vs_CK_Sardi group (**A**), YD_Longmu801_vs_CK_Longmu801 group (**B**), YD_Sardi_vs_CK_Sardi group (**C**), and YD_Longmu801_vs_YD_Sardi group (**D**). The DAMs were selected using a significance level of 0.05.

**Figure 4:**
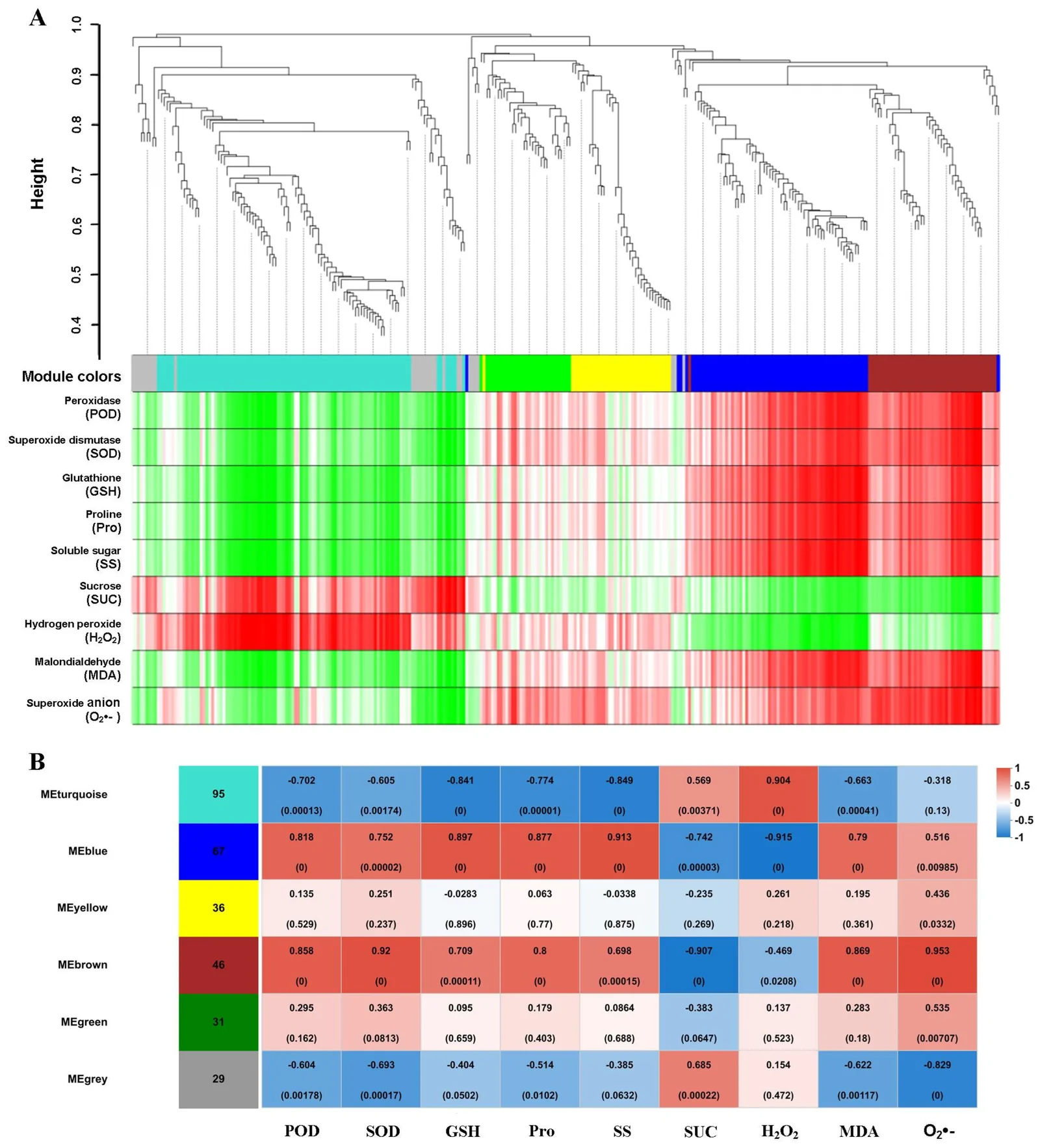
Correlation analysis and exploratory correlation-based module analysis between metabolite modules and physiological indicators. (**A**) Hierarchical clustering dendrogram (top) and module color assignment (middle), and the standardized abundance heatmap of physiological indices (bottom). The color scale from green to red represents the standardized values (Z-score) of these indices. The colored bars below the dendrogram represent different co-expression modules (e.g., MEturquoise, MEblue, etc.). (**B**) Heatmap of Pearson correlation coefficients between the identified modules and physiological indices. The numbers in each cell represent the correlation coefficient (top) and the corresponding *p*-value (bottom). The color gradient from blue (−1) to red (1) indicates negative and positive correlations, respectively.

**Figure 5:**
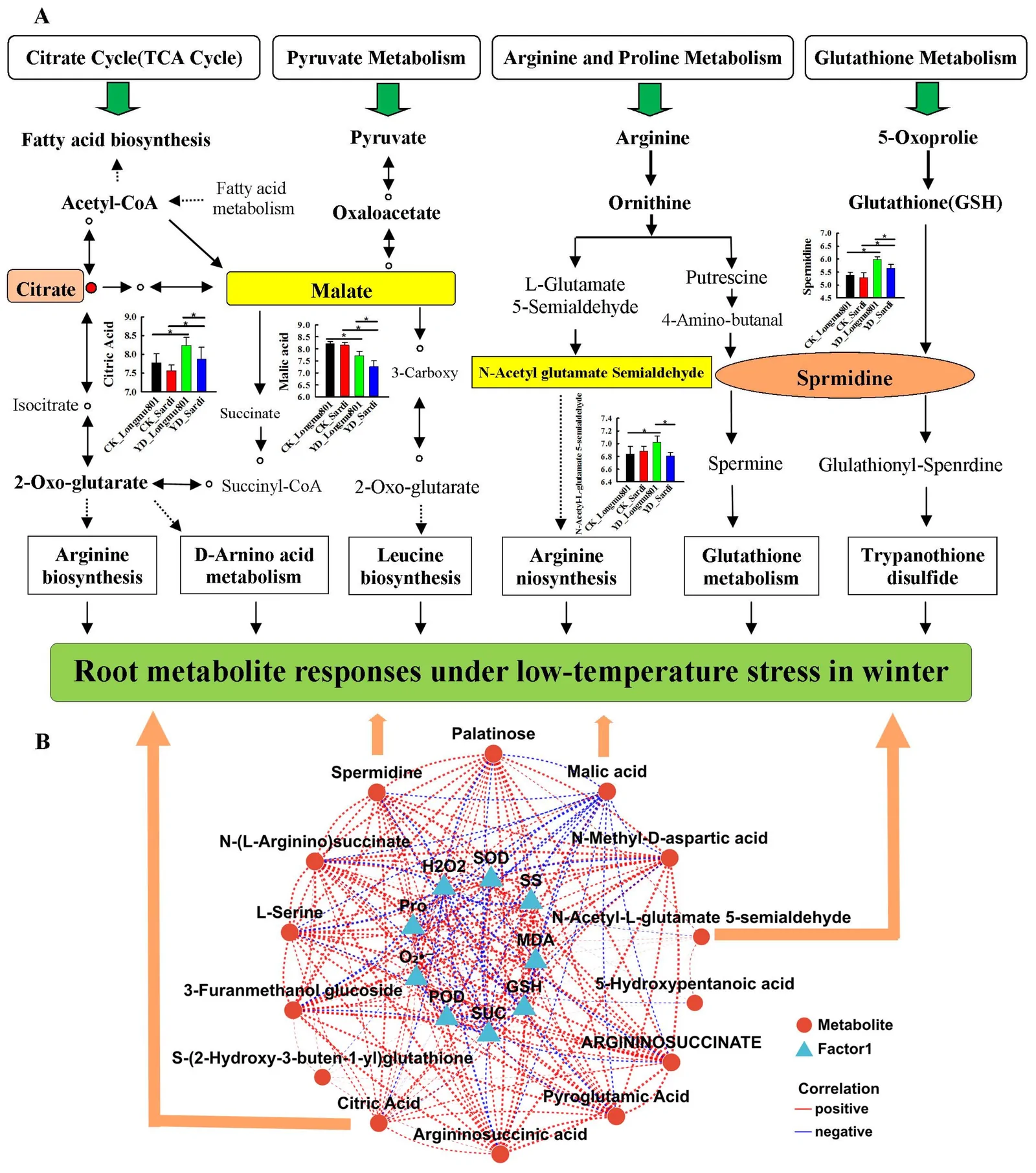
Metabolite pathway analysis. (**A**) Schematic diagram of the metabolic pathways. The arrows indicate the flow of metabolites. The embedded histograms show the relative contents of metabolites under different treatments. The *x*-axis represents the treatments, and the *y*-axis represents the relative metabolite content. Asterisks (*) denote significant differences among groups (*p* < 0.05). (**B**) Schematic diagram of the association between metabolites and physiological indicators. Nodes represent variables: red nodes outside the circles correspond to hub metabolites, whereas the triangular nodes correspond to physiological indicators. Edges (lines) represent the correlations between nodes: red dashed lines indicate positive correlations, and blue dashed lines indicate negative correlations.

## Data Availability

The original contributions presented in the study are included in the article/Supplementary Material. The detailed identification information and quantitative dataset of the metabolites are provided in Supplementary Tables S1 and S2. Further inquiries can be directed to the corresponding authors.

## Supplementary Materials

The following supporting information can be downloaded at: https://www.mdpi.com/article/10.3390/biology15212000/s1; Figure S1: Principal component analysis (PCA) of samples and Venn diagram of differential features for Longmu801 and Sardi varieties under different treatments; Figure S2: Hierarchical clustering heatmap of differential metabolites in Longmu801 and Sardi varieties under different treatments; Figure S3: Correlation analysis and hierarchical clustering heatmap of differential metabolites; Figure S4: Comparison of relative abundances of differential metabolites in Longmu801 and Sardi varieties under different treatments; Table S1: Summary of identification information and mass spectrometry characteristics for the principal metabolites; Table S2: Complete quantitative dataset of the 304 differentially accumulated metabolites (DAMs).
